# Supplementary figures and images for: Analysis of leaf morphology development-related genes and photosynthetic metabolic pathways in the transcriptomes of new leaves of Tea-Oil tree (Camellia oleifera ‘changlin53’)
Source: PLoS One. 2026 Jun 4;21(6):e0349387. doi: 10.1371/journal.pone.0349387 (PMC13235918; doi:10.1371/journal.pone.0349387)

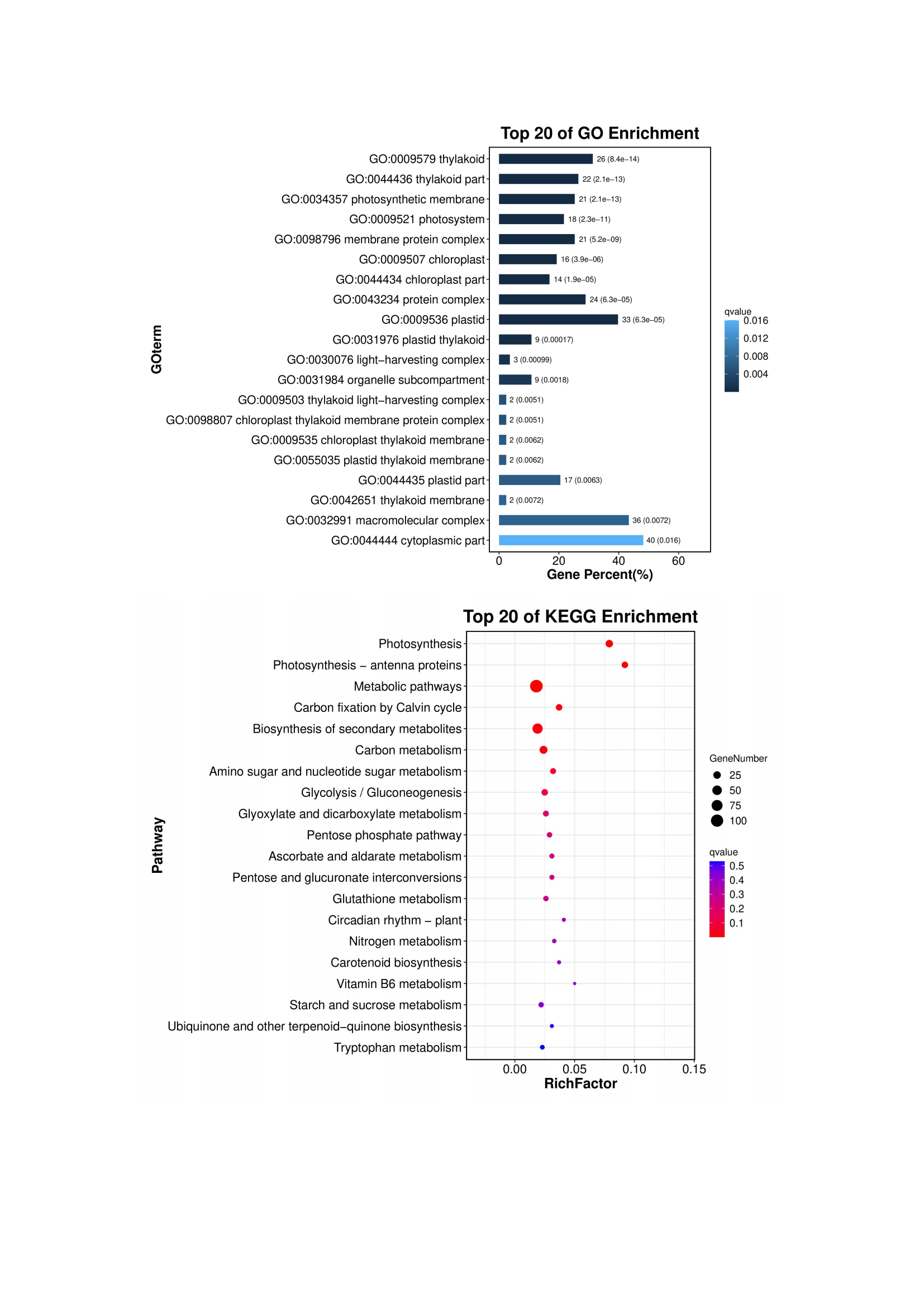

Supplement: S1 Fig — (JPG) [file pone.0349387.s003.jpg]
