# Supplementary material for: Analysis of leaf morphology development-related genes and photosynthetic metabolic pathways in the transcriptomes of new leaves of Tea-Oil tree (Camellia oleifera ‘changlin53’)
Source: PLoS One. 2026 Jun 4;21(6):e0349387. doi: 10.1371/journal.pone.0349387 (PMC13235918; doi:10.1371/journal.pone.0349387)
Supplement: Attachment — Submitted filename: comments.docx [file pone.0349387.s005.docx]

This study combined morphological and anatomical observations with multi-stage transcriptome sequencing to analyze the key regulatory networks underlying leaf functional maturation in *Camellia oleifera* 'Changlin 53'. However, the manuscript contains multiple issues with figure presentation and description. There are still some issues that need be improved and revised.

1. In Figure 1, the leaf buds are shown without a scale bar, making it difficult for readers to assess their actual size. It is recommended to include an appropriate scale bar or indicate magnification to allow for accurate interpretation of the morphological features.
2. The results presented in Figures 2 and 3 are essentially part of a single logical dataset. It is recommended to merge them into one figure (as Figure 2) to improve the coherence of data presentation. In the corresponding text, the authors should refer precisely to the specific panels (e.g., “Figure 3a”) rather than using a broad reference such as “Figures 2–3” to ensure accurate correspondence between the narrative and the visual data.
3. In the “Study of new growth pattern” section the manuscript states:
   “The results of Figures 2-2, 2-3, and 2-4 show that chlorophyll A content in new leaves during oil tea growth exhibited a similar pattern, indicating that chlorophyll A content can be used to evaluate oil tea growth.”
   The reference to “Figures 2-2, 2-3, and 2-4” is unclear. I could not find a figure labelled “Fig. 2-2” in the file; it is ambiguous whether the authors mean “Figs. 2–4”, “Figs. 2-2/2-4”, or specific subpanels (e.g., Fig. 2a, Fig. 2b). Please carefully check and correct all figure citations in the text so that each in-text reference matches an existing figure or panel.
4. In the “Sequencing quality analysis” section, the description of the results should explicitly indicate that the data are derived from Table 2-1. However, the current table numbering (Table 2-1) is inconsistent with standard journal formatting. It is recommended to simplify it to “Table 1” (Tab. 1) and to ensure consistent numbering throughout the manuscript. Additionally, please make sure that all references to tables in the text correspond correctly to the actual table numbers.
5. In the *Functional Annotation of Unigenes* section, the scientific (Latin) names of species are not italicized. According to standard biological nomenclature rules, all genus and species names should be presented in italics (e.g., *C. oleifera*). Please carefully revise this section to ensure all Latin names are correctly formatted.
6. In the *Sample relationship analysis* section, the text refers to “(Figure 2-1)”; however, this figure does not appear to correspond to the numbering in the submitted manuscript. Please carefully verify all figure citations to ensure that each in-text reference accurately matches the correct figure or panel. Consistent and correct figure numbering is essential for clarity and to avoid confusion for readers.
7. There are two figures both labeled as “Figure 6” in the manuscript. Please carefully check the figure numbering throughout the manuscript to ensure that each figure has a unique number and that in-text citations correspond correctly to the figures.
   Additionally, the legend of the second Figure 6 refers to panels “a” and “b”, but these subpanels are not present in the figure itself. Please verify that all figure panels are correctly displayed and that the legends accurately describe the figure content. Proper alignment between figures, subpanels, and captions is essential for clarity and accurate interpretation.
8. There are numerous instances of mismatched figure numbers and in-text citations throughout the manuscript. Please carefully verify all figures, subpanels, and references to ensure consistency and correct alignment.
9. In the *Plant Growth and Sample Collection* section, the coordinates are not correctly formatted.
10. In the Discussion section, “SAM19” appears, where “19” refers to a reference. According to standard journal conventions, the full reference should be provided when it is first mentioned, and subsequent mentions may use the abbreviation. Please carefully check the manuscript for similar instances where abbreviations or reference numbers are used at first mention, and ensure all follow proper formatting rules.
11. In the Methods section, the manuscript does not clearly indicate the developmental stage or sampling time corresponding to the samples labeled NLⅢ, NLⅣ, and NLⅤ. Please specify the exact stage or time point for each sample to ensure that the experimental design is fully reproducible and interpretable.
12. In section 4.6, the expression “2 −Ct” appears incorrectly formatted with respect to superscripts and subscripts. In standard qPCR notation, it should be written as “2^−ΔΔCt^ ” or the appropriate formula intended. Please carefully check the entire manuscript for similar issues of superscript and subscript misusage to ensure all formulas and gene expression notations are correctly presented.
