# Supplementary material for: Analysis of leaf morphology development-related genes and photosynthetic metabolic pathways in the transcriptomes of new leaves of Tea-Oil tree (Camellia oleifera ‘changlin53’)
Source: PLoS One. 2026 Jun 4;21(6):e0349387. doi: 10.1371/journal.pone.0349387 (PMC13235918; doi:10.1371/journal.pone.0349387)
Supplement: Attachment — Submitted filename: Response to Reviewers.docx [file pone.0349387.s006.docx]

Please use the space provided to explain your answers to the questions above. You may also include additional comments for the author, including concerns about dual publication, research ethics, or publication ethics. (Please upload your review as an attachment if it exceeds 20,000 characters)

Reviewer #1: This study combined morphological and anatomical observations with multi-stage transcriptome sequencing to analyze the key regulatory networks underlying leaf functional maturation in Camellia oleifera 'Changlin 53'. However, the manuscript contains multiple issues with figure presentation and description. There are still some issues that need be improved and revised.

1.In Figure 1, the leaf buds are shown without a scale bar, making it difficult for readers to assess their actual size. It is recommended to include an appropriate scale bar or indicate magnification to allow for accurate interpretation of the morphological features. Answer:Modified

2.The results presented in Figures 2 and 3 are essentially part of a single logical dataset. It is recommended to merge them into one figure (as Figure 2) to improve the coherence of data presentation. In the corresponding text, the authors should refer precisely to the specific panels (e.g., “Figure 3a”) rather than using a broad reference such as “Figures 2–3” to ensure accurate correspondence between the narrative and the visual data.

Answer:Modified

3.In the “Study of new growth pattern” section the manuscript states:

“The results of Figures 2-2, 2-3, and 2-4 show that chlorophyll A content in new leaves during oil tea growth exhibited a similar pattern, indicating that chlorophyll A content can be used to evaluate oil tea growth.”

The reference to “Figures 2-2, 2-3, and 2-4” is unclear. I could not find a figure labelled “Fig. 2-2” in the file; it is ambiguous whether the authors mean “Figs. 2–4”, “Figs. 2-2/2-4”, or specific subpanels (e.g., Fig. 2a, Fig. 2b). Please carefully check and correct all figure citations in the text so that each in-text reference matches an existing figure or panel.

Answer:Modified

4.In the “Sequencing quality analysis” section, the description of the results should explicitly indicate that the data are derived from Table 2-1. However, the current table numbering (Table 2-1) is inconsistent with standard journal formatting. It is recommended to simplify it to “Table 1” (Tab. 1) and to ensure consistent numbering throughout the manuscript. Additionally, please make sure that all references to tables in the text correspond correctly to the actual table numbers.

Answer:Modified

5.In the Functional Annotation of Unigenes section, the scientific (Latin) names of species are not italicized. According to standard biological nomenclature rules, all genus and species names should be presented in italics (e.g., C. oleifera). Please carefully revise this section to ensure all Latin names are correctly formatted.

Answer:Modified

6.In the Sample relationship analysis section, the text refers to “(Figure 2-1)”; however, this figure does not appear to correspond to the numbering in the submitted manuscript. Please carefully verify all figure citations to ensure that each in-text reference accurately matches the correct figure or panel. Consistent and correct figure numbering is essential for clarity and to avoid confusion for readers.

Answer:Modified

7.There are two figures both labeled as “Figure 6” in the manuscript. Please carefully check the figure numbering throughout the manuscript to ensure that each figure has a unique number and that in-text citations correspond correctly to the figures.

Additionally, the legend of the second Figure 6 refers to panels “a” and “b”, but these subpanels are not present in the figure itself. Please verify that all figure panels are correctly displayed and that the legends accurately describe the figure content. Proper alignment between figures, subpanels, and captions is essential for clarity and accurate interpretation.

Answer:Modified

8.There are numerous instances of mismatched figure numbers and in-text citations throughout the manuscript. Please carefully verify all figures, subpanels, and references to ensure consistency and correct alignment.

Answer:Modified

9.In the Plant Growth and Sample Collection section, the coordinates are not correctly formatted.

Answer:Modified

10.In the Discussion section, “SAM19” appears, where “19” refers to a reference. According to standard journal conventions, the full reference should be provided when it is first mentioned, and subsequent mentions may use the abbreviation. Please carefully check the manuscript for similar instances where abbreviations or reference numbers are used at first mention, and ensure all follow proper formatting rules.

Answer:Modified

11.In the Methods section, the manuscript does not clearly indicate the developmental stage or sampling time corresponding to the samples labeled NLⅢ, NLⅣ, and NLⅤ. Please specify the exact stage or time point for each sample to ensure that the experimental design is fully reproducible and interpretable.

Answer:Modified

12.In section 4.6, the expression “2 −Ct” appears incorrectly formatted with respect to superscripts and subscripts. In standard qPCR notation, it should be written as “2−ΔΔCt ” or the appropriate formula intended. Please carefully check the entire manuscript for similar issues of superscript and subscript misusage to ensure all formulas and gene expression notations are correctly presented.

Answer:Modified

Reviewer #2: This study presented the first systematic elucidation of the molecular mechanisms underlying the leaves development in Camellia oleifera. They found that the enhancement of photosynthesis is closely coordinated with carbon and nitrogen metabolism during leaf maturation by using transcriptome sequencing. This result offers molecular insights for improving yield and quality. However, there are several limitations in the experimental design, results, and derivation of conclusions. The following points require further clarification or supplementation to strengthen the overall validity and impact of the study.

1.As for transcriptome data, although a number of DEGs were identified, key regulatory genes or core modules (for example, transcription factors, TFs) were not highlighted. WGCNA is helpful for in-depth analysis of the “photosynthesis–metabolism” regulatory network.

Answer:Modified

2.In Figures 7-10, the authors presented GO and KEGG enrichment analysis at the surface level. Visualizing key DEGs or gene–pathway association networks would improve interpretability.

Answer:Modified

3.Although numerous pathways (for example, PAL, 4CL, FLS) were presented, logically, it is unclear how different pathways (photosynthesis, hormones, secondary metabolism) change during different phases. The related contents should be added in the discussion section.

Answer:Modified

4. The authors should emphasize the comparison of C. oleifera leaf development and other woody oil crops (for example, Vernicia fordii, oil palm) in addition to tomato and then discuss whether C. oleifera exhibits unique photosynthetic regulation or metabolic advantages.

Answer:Modified

5. The text need to be edited by a native English speaker.

Answer:Modified

Reviewer #3: This manuscript investigates the gene expression dynamics and photosynthetic metabolic pathways during new leaf development in Camellia oleifera'Changlin53' by integrating morphological observations and transcriptomic analyses. The study reveals critical mechanisms underlying leaf functional maturation, offering theoretical insights for improving oil tea yield through breeding. However, significant revisions are required to address language, structural, and methodological issues before publication. Specifically, I have the following questions:

1. There are grammatical errors and unclear expressions in the Abstract and main text. For example, inconsistent usage of 'oil tea' and 'tea oil', and a spelling error in 'photosynthesis-mochromism'. It is recommended that the manuscript undergo a thorough revision by native English speakers or professional editors, with a focus on simplifying sentence structures and correcting grammatical inaccuracies;

Answer:Modified

2. The qPCR data, although mentioning three biological replicates, were not presented in the figures. It is recommended to supplement statistical analyses (e.g., ANOVA) to validate the reliability of the results;

Answer:Modified

3. The figure captions are inconsistent with the main text. For example, 'Figure 2-1' lacks a corresponding figure caption. 'Figure 1' is not cited in the text and should be either removed or properly referenced. For the figure image, it is recommended to add small icons to highlight key features;

Answer:Modified

4. The Discussion section is somewhat superficial and needs to be rewritten for greater depth and analytical rigor;

Answer:Modified

5. The manuscript lacks a description of the chlorophyll content determination methods;

Answer:Modified

6. Some pictures are suggested to be reorganized for presentation. The pictures are too simple and unaesthetic;

Answer:Modified

7. In Figure 8 (Photosynthetic Signaling Pathways), the annotations for gene upregulation/downregulation lack clarity, and there is no direct linkage between these molecular changes and physiological parameters (e.g., chlorophyll content, photosynthetic efficiency). Integration of multi-omics datasets (physiological and molecular) is strongly recommended to strengthen mechanistic interpretations and experimental validity.；

Answer:Modified

8. It is recommended to supplement the determination of photosynthetic parameters, as chlorophyll content may not necessarily correlate with photosynthetic efficiency.;

Answer:This part of the trial will be supplemented in subsequent studies.

9. The conclusion section asserts that 'there are shared regulatory mechanisms with processes such as seed maturation', yet lacks comparative data across different organs. It is advisable to either refine the scope of conclusions or provide a critical discussion of the study's limitations in organ-specific regulatory networks;

Answer:Modified

10. The Results section lacks logical flow and is disorganized. It is recommended to reorganize the content following the sequence: morphological changes → transcriptomic dynamics → pathway analysis;

Answer:Modified

11. The association between ABA and JA signaling pathways and leaf stress resistance requires further experimental validation; additional data support is recommended;

Answer:Modified

12. The reference formatting is inconsistent, and citation information is incomplete in some entries. It is recommended to revise them in accordance with the target journal's guidelines;

Answer:Modified

14. qRT-PCR primer sequences are missing;

Answer:Modified

15. RNA-Seq library construction workflow is missing.

Answer:Modified
