# Supplementary material for: Analysis of leaf morphology development-related genes and photosynthetic metabolic pathways in the transcriptomes of new leaves of Tea-Oil tree (Camellia oleifera ‘changlin53’)
Source: PLoS One. 2026 Jun 4;21(6):e0349387. doi: 10.1371/journal.pone.0349387 (PMC13235918; doi:10.1371/journal.pone.0349387)
Supplement: Attachment — Submitted filename: Response to Reviewers.doc [file pone.0349387.s008.doc]

There are still some issues that need be improved and revised.

1. Please note that after the first full introduction of the species as "*Camellia oleifera*" , its subsequent mentions in the text, figures, and tables should be abbreviated to "*C. oleifera*" for consistency and in accordance with standard botanical nomenclature conventions.

Answer:Modified

2. Please resolve all instances of the citation error "Error! Reference source not found" (e.g., in Plant Growth and Sample Collection, line 6). Ensure every citation is correctly linked to its reference list entry.

Answer:Modified

3. In the Introduction section (Paragraph 2, Line 9), there is a citation formatting error: "ecological restoration ^11,1212^". The reference number "12" appears to be duplicated. Please systematically check all in-text citations for similar repetitive or incorrect numbering and ensure they are correctly linked to the reference list.

Answer:Modified

4. Figure 2 contains unreadable or unknown content, as highlighted in the attached screenshot (red markings).

Answer:Modified

5. The manuscript contains sporadic Chinese characters (e.g., full-width punctuation such as “：”, in the caption of Figure 2 ) and instances of missing spaces between words. These formatting errors detract from the text's professionalism and readability. Please conduct a thorough, line-by-line review to correct these issues and ensure the manuscript adheres to standard English formatting conventions.

Answer:Modified

6. The caption for Figure 5 is incomplete.

Answer:Modified

7. In the section "KEGG enrichment of DEGs," the phrase "as illustrated in Figure 8" appears twice in close proximity, creating unnecessary redundancy and disrupting the narrative flow. Please revise the text to state the finding concisely and cite the figure only once, typically at the most logical point where the data is first summarized or In the Methods section, you state that qRT-PCR experiments were performed with three replicates. However, the corresponding Figure 11 does not display any error bars or measures of variability (e.g., standard deviation or standard error of the mean).

Answer:Modified

8. In the Discussion section, The phrase "SAM 23" is ambiguous. Clarify whether "23" is a referenceor part of a name ?

Answer:Modified
