# Supplementary material for: Analysis of leaf morphology development-related genes and photosynthetic metabolic pathways in the transcriptomes of new leaves of Tea-Oil tree (Camellia oleifera ‘changlin53’)
Source: PLoS One. 2026 Jun 4;21(6):e0349387. doi: 10.1371/journal.pone.0349387 (PMC13235918; doi:10.1371/journal.pone.0349387)
Supplement: Attachment — Submitted filename: comments.doc [file pone.0349387.s009.doc]

Dear authors,

Camellia oleifera is an important woody edible oil tree species in China. This paper “Analysis of leaf morphology development-related genes an d photosynthetic metabolic pathways in the transcriptomes of new leaves of Tea-Oil tree (Camellia oleifera ‘changlin5 3’)” was good. I recommented this paper to accept.
